# Bringing back ‘significance’ to MGUS

**DOI:** 10.1093/rheumatology/keag115

**Published:** 2026-03-15

**Authors:** Koushan Kouranloo, Pasan Serasinghe, Majid Kazmi, Sajitha Sachchithanantham, Neeraj Kohli, Matthew Streetly, Emma Benton, David D’Cruz

**Affiliations:** Department of Rheumatology, King’s College Hospital, London, UK; Department of Medical Education, King’s College London, London, UK; Department of Rheumatology, Guy’s & St Thomas’ Hospital NHS Foundation Trust, London, UK; Clinical Haematology, Guys Hospital, Guys and St Thomas’ NHS Foundation Trust, London, UK; Clinical Haematology, Guys Hospital, Guys and St Thomas’ NHS Foundation Trust, London, UK; Clinical Haematology, Guys Hospital, Guys and St Thomas’ NHS Foundation Trust, London, UK; Clinical Haematology, Guys Hospital, Guys and St Thomas’ NHS Foundation Trust, London, UK; Department of Haematological Medicine, Kings College Hospital, London, UK; Department of Dermatology, Guy’s & St Thomas’ Hospital NHS Foundation Trust, London, UK; Department of Rheumatology, Guy’s & St Thomas’ Hospital NHS Foundation Trust, London, UK

Rheumatology key message

- Monoclonal Gammopathy of Undetermined Significance (MGUS) terminology now reflects organ involvement and should be considered in patients presenting with presumed vasculitis.

Dear Editor, A 48-year-old lady of Afro-Caribbean ancestry, with hypertension and glucose-6-phosphate dehydrogenase (G6PD) deficiency presented with pruritis, predominantly affecting the face and limbs, and an urticarial rash, worse on cold exposure (Fig. 1A). No other relevant signs or symptoms, including autoimmune features, were noted. There was no relevant family history, and no history of thrombotic events or pregnancy losses. She was a registered nurse, non-smoker and did not consume any alcohol. She was given an initial diagnosis of lichen planus and commenced on corticosteroids.

**Figure 1:**
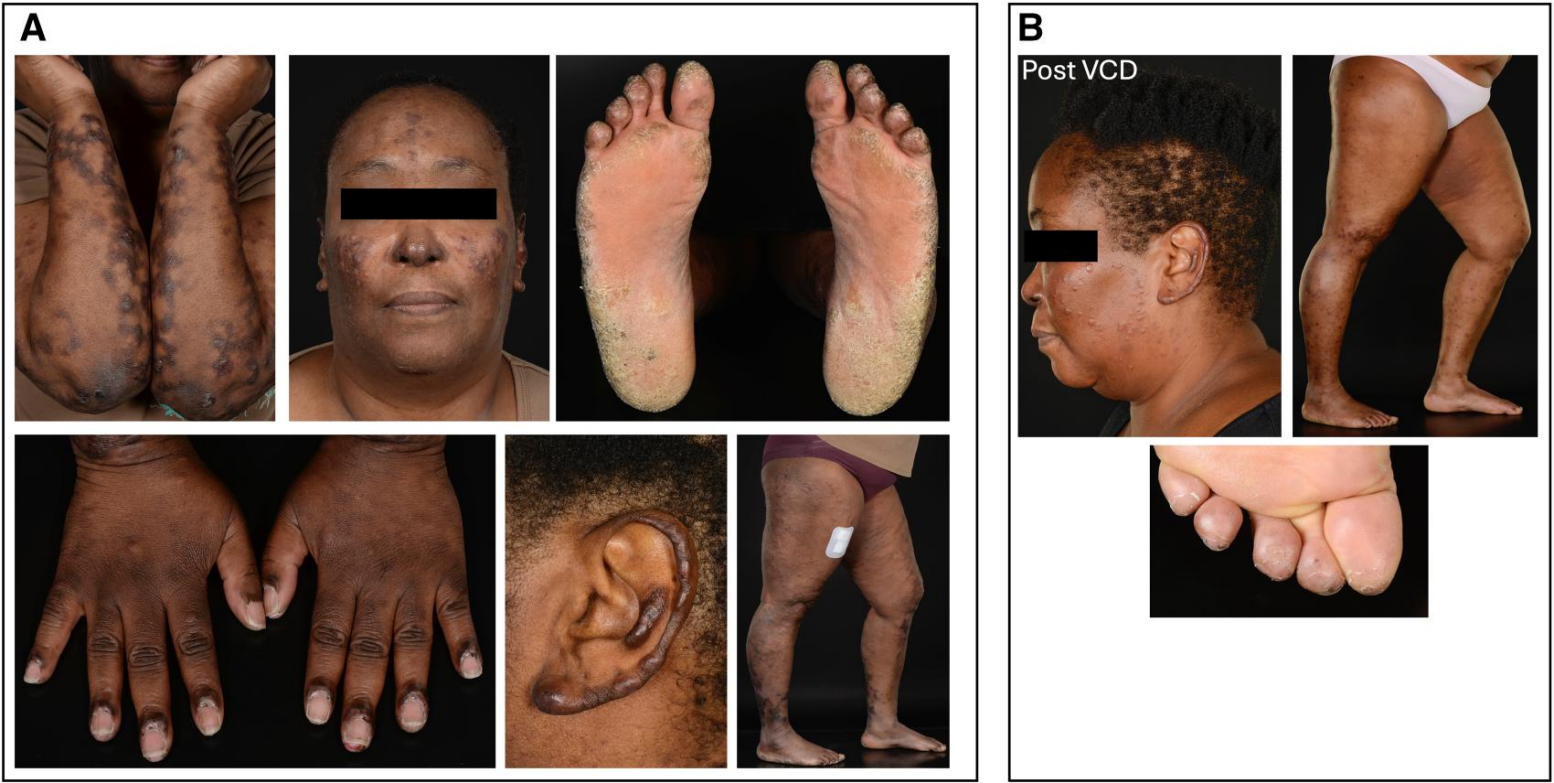
Clinical manifestations at initial presentation, prior to bortezomib (Velcade), cyclophosphamide, dexamethasone (VCD) therapy (**A**) and clinical presentation following monoclonal gammopathy of clinical significance (MGCS) diagnosis and VCD therapy (**B**)

Two years later, due to secondary steroid failure, she underwent a skin biopsy, which revealed urticarial vasculitis. She was subsequently trialled on multiple disease-modifying agents including azathioprine, hydroxychloroquine and mycophenolate mofetil, as well as colchicine, all stopped due to primary failure or adverse effects. She subsequently lost response to plasmapheresis. Dapsone was considered but never tried due to G6PD deficiency. Three years later, she had a 4-month course of methotrexate 20 mg weekly with ciclosporin 150 mg daily and 5 mg prednisolone. This resulted in improvement of the rash in her lower limbs, but not her upper limbs or face. By this point, her affected skin was profoundly itchy, burning and painful, accompanied by hair-loss and scarring. A repeat skin biopsy was consistent with leukocytoclastic vasculitis. Numerous neutrophils and neutrophilic debris with focal extravasated erythrocytes were noted; immunofluorescence was negative. Serum immunoglobulin levels and a full autoimmune connective tissue disease screen were normal.

She was subsequently referred to rheumatology, having been managed by dermatology and immunology. She had developed joint pains in the hips, ankles, fingers and toes, recurrent nosebleeds and shortness of breath on exertion. She was unable to reduce her prednisolone to below 20 mg daily. A single digital ulcer was noted without Raynaud’s features. Nailfold capillaroscopy showed increased tortuosity and dilated capillary loops but no vasculitis.

Examination revealed a pruritic rash over her hands in a Gottron’s distribution and periungual pigmentation. Extensive urticarial vasculitic lesions were seen over her arms, legs, face and ears (Fig. 1A). She had a peripheral sensory neuropathy in a glove and stocking distribution to the levels of her elbows and ankles without a motor deficit. Investigations showed mildly raised inflammatory markers (ESR 34 mm/h, CRP 9 mg/l) and raised IgA levels (4.58 g/l) with paraproteins. The haematology and biochemistry results were unremarkable, and connective tissue screen, including ANA, ENA, dsDNA, MPO/PR3 ANCA, rheumatoid factor and cryoglobulins, continued to be negative.

Unable to taper her steroids, and with a worsening urticarial vasculitis, sensory loss and new extensive digital vasculitic infarcts, she was commenced on intravenous cyclophosphamide (EuroLupus regimen), with pulsed methylprednisolone. At this time, she had a Birmingham Vasculitis Activity Score of 17. There was a temporary improvement in her symptoms, but after a week, the rash returned. She resumed high-dose oral prednisolone and remained symptomatic. In light of the persistently raised IgA, and presence of IgA kappa paraprotein, she was referred to haematology, with concerns that her presentation may be part of a paraneoplastic syndrome, related to her monoclonal protein.

Almost 18 months after receiving cyclophosphamide, she received a diagnosis of monoclonal gammopathy of undetermined significance (MGUS), following a bone marrow biopsy showing 5–8% IgA clonal plasma cells with normal serum free light chains. Paraprotein level was too small to quantify (<2 g/l), with no evidence of myeloma. There was no evidence of amyloid present on Congo Red stain. A PET CT scan demonstrated FDG-avid cutaneous lesions but no focal bone lesions or underlying neoplasia. She continued to have a persistent treatment-resistant vasculitis, on varying levels of prednisolone but never below 10 mg. A diagnosis of urticarial vasculitis related to IgA MGUS was considered likely. However, after a further year of no response to treatment for her vasculitis, she was commenced on plasma cell directed therapy with bortezomib (Velcade™), cyclophosphamide, dexamethasone (VCD) treatment.

The patient had an excellent response with just one cycle of treatment, after which her monoclonal protein was no longer detectable by protein electrophoresis, resulting in significant improvements in the vasculitic lesions within 24 h (Fig. 1B). She completed six cycles of VCD in total and continued to make an excellent improvement in all aspects of her disease.

This case demonstrates the cautious use of terminology of MGUS, which has now evolved to reflect a greater emphasis on its clinical implications, particularly regarding organ involvement. Previously viewed primarily as benign or a precursor to plasma cell disorders, MGUS is being recognized to have a broader spectrum of presentations, including where monoclonal proteins can directly cause end organ sequelae, increasingly termed monoclonal gammopathy of clinical significance (MGCS) [1, 2]. MGCS ought to be carefully considered when caring for patients with diagnosis of MGUS combined with an otherwise idiopathic leukocytoclastic vasculitis. This shift highlights the importance of recognizing MGUS in the presence of paraprotein that is not responding to standard treatment, to enable early involvement of a multidisciplinary team for closer monitoring and early intervention with plasma cell targeting therapy to prevent long-term organ dysfunction.

## Data Availability

All data available upon reasonable request.
